# Immunological mechanisms in Meniere’s disease

**DOI:** 10.3389/fimmu.2025.1639916

**Published:** 2025-08-27

**Authors:** Vincent G. Yuan, Anping Xia, Peter L. Santa Maria

**Affiliations:** Department of Otolaryngology-Head and Neck Surgery, University of Pittsburgh Medical Center, Pittsburg, PA, United States

**Keywords:** Meniere’s disease (MD), immune dysregulation, endolymphatic hydrops, inner ear disorders, innate immunity, adaptive immunity, immunotherapy, precision medicine

## Abstract

Meniere’s disease (MD) is a chronic, relapsing inner ear disorder characterized by episodes of vertigo, fluctuating sensorineural hearing loss, tinnitus, and aural fullness. Although its etiology has long remained elusive, accumulating evidence implicates immune dysregulation as a central contributor to disease pathogenesis, particularly in patients who do not respond to standard therapies. This review synthesizes current insights into the immunopathological mechanisms underlying MD, focusing on the roles of both innate and adaptive immune cells—including macrophages, dendritic cells, T cells, and B cells—in promoting inflammation, endolymphatic hydrops, and sensory dysfunction. We examine the contribution of these immune cells to tissue damage, along with the roles of cytokine signaling and immune complex deposition. Emerging immunotherapies, including corticosteroids, biologics, and inflammasome inhibitors, are evaluated for their therapeutic potential. The review also highlights promising directions in precision medicine, such as immune profiling, biomarker discovery, and stratified clinical trials aimed at personalizing treatment. By integrating recent immunological advances with clinical management strategies, we underscore the potential of immune guided approaches to transform the diagnosis, treatment, and long-term care of patients with MD.

## Introduction

Meniere’s Disease (MD) is a long-term and disabling inner ear condition marked by recurring episodes of vertigo, variable hearing loss, ringing in the ears (tinnitus), and a feeling of fullness in the affected ear (1–3). First described by Prosper Ménière in 1861, the disease remains poorly understood, with patients enduring unpredictable symptom flare-ups that can severely impair their quality of life (4). While MD typically begins as a unilateral condition, bilateral involvement may develop in a significant subset of patients over time. The most distressing symptom, vertigo, can last from 20 minutes to several hours, often accompanied by sensorineural hearing loss that may eventually become permanent (5).

**Figure 1:**
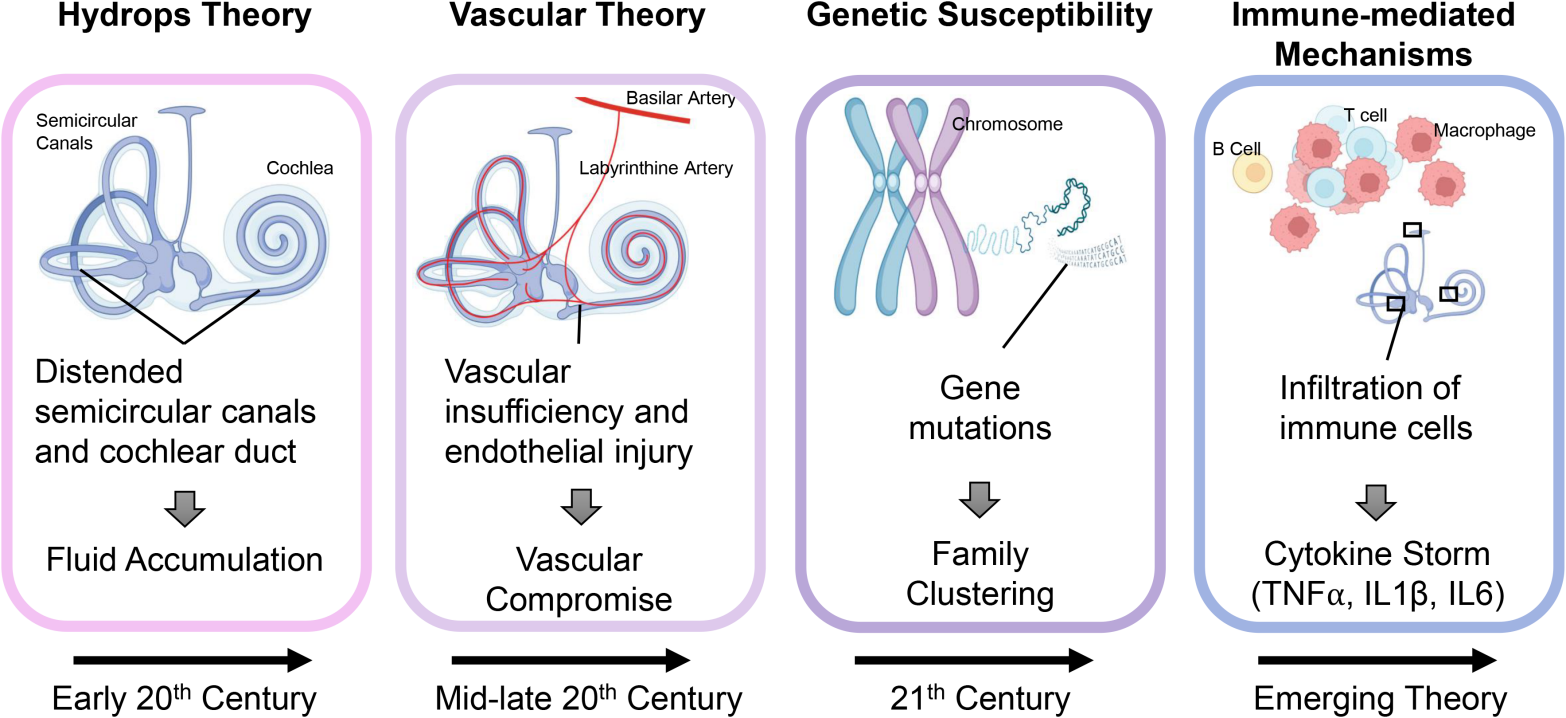
Evolving theories on the pathogenesis of Meniere’s disease. Over time, the understanding of MD pathogenesis has progressed through several key theories. In the early 20th century, MD was attributed to endolymphatic hydrops, based on the observation of distended semicircular canals and cochlear ducts suggesting abnormal fluid accumulation. By the mid to late 20th century, vascular insufficiency and endothelial injury were proposed as primary causes, leading to compromised blood flow and secondary hydrops. In the 21st century, genetic susceptibility emerged, with familial studies identifying mutations linked to inner ear dysfunction. More recently, immune-driven processes have garnered increased attention, evidenced by the presence of immune cell infiltration and heightened levels of proinflammatory cytokines such as TNF-α, IL-1β, and IL-6, which play a role in inner ear injury and the development of hydrops.

The etiology of MD remains elusive, with numerous theories proposed to explain its pathophysiology (**Figure 1**). Traditionally, MD has been associated with endolymphatic hydrops, a condition characterized by the abnormal accumulation of endolymph within the cochlea and vestibular system (6–8). However, emerging research suggests that MD may involve additional contributing factors, including genetic predisposition, vascular abnormalities, and autoimmune mechanisms (9–11). Notably, growing evidence suggests that immune dysregulation plays a central role in driving the onset and advancement of MD. Both innate and adaptive immune responses appear to contribute to tissue damage and chronic inflammation within the inner ear, exacerbating the disease (12–16).

This review aims to explore the immunological mechanisms underlying MD, focusing on how innate and adaptive immune responses contribute to disease mechanisms, while also emphasizing the promise of therapies that modulate immune responses in treating this complex and poorly understood condition.

## Pathophysiology of Meniere’s disease: a historical perspective

For much of its history (**Figure 1**), MD has been understood primarily through the lens of structural abnormalities within the inner ear (17). The most widely accepted theory, proposed in the early 20th century, is the hydrops theory, which attributes MD symptoms to endolymphatic hydrops — an abnormal fluid buildup (18, 19). The resulting labyrinthine distention disrupts vestibular and auditory function, producing episodic vertigo, tinnitus, and hearing loss (20, 21). Histological studies have confirmed the presence of endolymphatic hydrops in many MD patients, cementing this theory as a cornerstone of MD pathophysiology for decades (17).

While the hydrops theory remains foundational, it does not fully explain the heterogeneity of the disease or the variable response to treatments aimed at reducing fluid accumulation. In light of these limitations, alternative theories have emerged. Vascular theories suggest that MD may result from microvascular dysfunction, leading to ischemia within the inner ear (11, 19, 22). Supporting this idea is the observation that MD frequently occurs alongside other vascular conditions, such as migraine and hypertension, suggesting a potential connection between impaired blood circulation and inner ear dysfunction (23, 24). Similarly, researchers have investigated genetic predisposition, noting familial clustering and specific gene variants that may contribute to a heightened susceptibility to MD, indicating a hereditary influence in some cases (10, 25).

**Figure 2:**
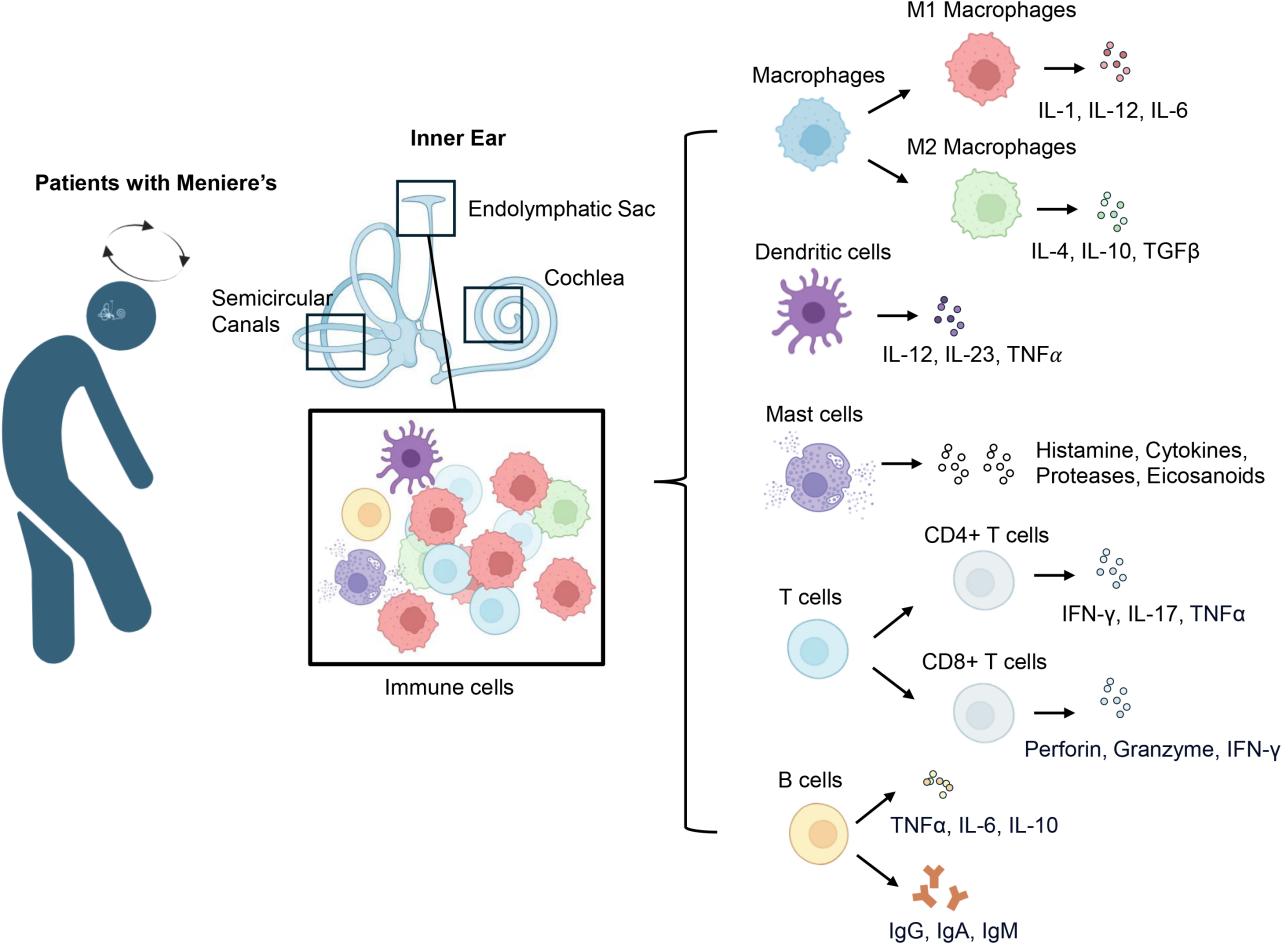
Immune cell infiltration and cytokine profiles in Meniere’s disease. In patients with MD, diverse immune cells infiltrate the vestibular system, including the endolymphatic sac, semicircular canals, and cochlea. The predominant immune cell types include macrophages, dendritic cells, mast cells, T cells, and B cells. Macrophages are categorized into pro-inflammatory M1 and anti-inflammatory M2 phenotypes: M1 macrophages release cytokines including IL-1, IL-6, and IL-12, whereas M2 macrophages generate anti-inflammatory mediators such as IL-4, IL-10, and TGF-β. Dendritic cells play a central role in antigen presentation and release cytokines such as IL-12, IL-23, and TNF-α. Mast cells are highly responsive to various stimuli and release a broad array of mediators, including histamine, cytokines, proteases, and eicosanoids. T cells mainly consist of CD4^+^ and CD8^+^ populations; CD4^+^ T cells perform both regulatory and effector roles by secreting cytokines such as IFN-γ, IL-17, and TNF-α, while CD8^+^ T cells promote tissue injury through the release of perforin, granzyme, and IFN-γ. B cells in MD produce antibodies including IgG, IgA, and IgM and release cytokines like TNF-α, IL-6, and IL-10, which may influence immune regulation and play a role in disease advancement.

The contribution of immune mechanisms to the onset and progression of MD has attracted growing interest in recent years. Studies have identified immune-mediated mechanisms, including autoimmune responses and chronic inflammation, as potential contributors to the disease (9, 26). This evolving understanding of immune involvement challenges earlier theories that focused solely on mechanical or vascular factors. Findings such as immune cell infiltration, elevated cytokine levels, and circulating autoantibodies in MD patients support a role for immune dysregulation (**Figure 2**) (27–29). MD is now recognized as a multifactorial disorder in which immune dysregulation synergizes with hydrops, vascular insufficiency, and genetic susceptibility (5).

## Innate and adaptive immune responses in Meniere’s disease

### Macrophages

Monocytes and macrophages are crucial innate immune cells within the inner ear that regulate tissue repair, immune surveillance, and inflammation. Histopathological studies have revealed macrophage infiltration in the endolymphatic sac of MD patients (30). Macrophages are likely recruited in response to damage-associated molecular patterns (DAMPs) released by stressed or necrotic inner ear cells. Upon activation via Toll-like receptors (TLRs), particularly TLR2 and TLR4, macrophages initiate proinflammatory signaling cascades (31). Multiplex immunoassays reveal elevated levels of monocyte-related cytokines in patients with MD (32–34), further implicating them in persistent inflammation and tissue remodeling.

Macrophages exhibit dynamic functional plasticity, shifting between pro-inflammatory and tissue-repairing states (35). M1 macrophages release pro-inflammatory cytokines, including TNF-α, IL-1β, and IL-6, which contribute to tissue damage (36). In contrast, M2 macrophages secrete IL-10 and TGF-β, which promote tissue repair and maintain homeostasis (37). In inner ear diseases, this balance appears disrupted, with evidence of a shift toward an M1-dominant phenotype (CD80+) over the M2 reparative phenotype (CD32+), especially in patients with elevated IL-1β levels (38–40). Elevated TNF-α, IL-1β, and IL-6 in MD induce oxidative stress that ultimately damages inner ear structures (27, 41, 42). Additionally, elevated levels of CCL2 (MCP-1) in MD have been shown to sustain chronic inflammation by recruiting monocytes to inner ear tissues (33, 43, 44). Elevated plasma CCL2 levels also correlate with both hearing thresholds and IL-6 levels in MD patients, further underscoring their pathogenic relevance (33). Pro-inflammatory cytokines also stimulate excessive extracellular matrix deposition, disrupting cochlear and vestibular function (45, 46). Beyond cytokines, macrophage-derived MMP-9 is elevated in MD and capable of degrading extracellular matrix components, with levels correlating with disease progression (47–49).

In addition to cytokine production, macrophages connect innate and adaptive immunity by acting as antigen-presenting cells (APCs). They present antigens via MHC molecules to T cells, shaping T helper (Th) cell responses (50). Elevated frequencies of CD4+ T helper cells during acute attacks suggest macrophage-driven activation of adaptive immunity (51). Macrophage-derived IL-23 promotes the development of Th17 cells, which secrete IL-17, an immune mediator associated with persistent inflammatory responses and autoimmune disorders (52). An imbalance between IL-17 and IL-10 has been observed in MD patients, further supporting a pathogenic Th17 signature (26). These findings highlight the complex immunological landscape of MD, in which macrophages play a pivotal role in both initiating and perpetuating inflammation. A deeper understanding of regulatory mechanisms that shape macrophage behavior in MD may offer therapeutic insights.

### Dendritic cells

Dendritic cells (DCs) are pivotal in host defense, bridging innate and adaptive immunity (53). DCs specialize in immune surveillance, detecting pathogens and danger signals and initiating adaptive responses (54). The role of dendritic cells remains largely unexamined in MD; however, emerging evidence indicates that dendritic cells could be instrumental in sustaining the chronic inflammation linked to endolymphatic hydrops (29).

Dendritic cells are extensively present throughout the body, including within the cochlear and vestibular tissues, where they act as vigilant immune sentinels (55, 56). In the inner ear, DCs are found in the cochlea and endolymphatic sac—key sites for both fluid regulation and immune activity (57, 58). The endolymphatic sac is particularly notable as a site of active immune surveillance, containing APCs such as DCs and macrophages (59). Histological and immunohistochemical analyses of inner ear tissues have identified cells expressing CD11c (60) and MHC class II (61), markers characteristic of dendritic cell populations (57). Increased expression of dendritic cell-related biomarkers has been detected in the endolymphatic sac of patients with MD, implying a potential involvement of dendritic cells in the underlying mechanisms of the condition (29, 62). They may be activated by endogenous signals from damaged cells or by external stimuli such as infection (63).

Once activated, dendritic cells mature and travel to nearby lymph nodes, where they display processed antigens for recognition by naïve T lymphocytes and upregulate co-stimulatory surface proteins, including CD80, CD86, and CD40 (54). In the context of MD, increased DC activity may result in the priming of autoreactive T cells, perpetuating chronic inflammation (29). The cytokine milieu produced by activated DCs in the inner ear supports a pro-inflammatory phenotype: IL-12 fosters Th1 differentiation and IFN-γ production, IL-23 expands Th17 cells, and TNF-α and IL-6 exacerbate local inflammation and disrupt the blood-labyrinth barrier (BLB) (57, 64, 65). This cascade of DC-driven immune responses may underline the recurrent symptoms of MD, such as sensorineural hearing loss and episodic vertigo (66, 67).

Dendritic cells also influence humoral immunity. By releasing cytokines like BAFF (B cell–activating factor), they support the survival of B lymphocytes and facilitate their maturation into antibody-secreting plasma cells (68, 69). In MD, increased B cell activity and autoantibody production have been documented in some patients, indicating that DC-mediated B cell stimulation may further contribute to disease progression (29). The resulting autoantibodies may aggravate tissue damage and sustain the inflammatory state.

### Mast cells

A history of allergic conditions is present in 58% of MD patients, highlighting a potential immunoallergic component (70). Mast cells, key effector cells in allergic responses, have been increasingly implicated in the pathophysiology of MD. Specifically, mast cells in the endolymphatic sac are of particular interest because the ES plays a central role in endolymph homeostasis and immunoregulation (71, 72). In mice and rats, mast cells were observed around Reissner’s membrane on the scala vestibuli side, but were not detected in or near the organ of Corti (73).

Despite this distribution, mast cells exert dual roles in immune protection and inflammatory processes by releasing histamine, proinflammatory cytokines (TNF-α, IL-6), enzymes (tryptase, chymase), and lipid-derived mediators called eicosanoids (74–76). These mediators can alter vascular permeability, modulate epithelial integrity, and promote leukocyte recruitment (77). In the context of MD, mast cell degranulation in the ES and other cochlear structures may lead to breakdown of the blood-labyrinth barrier and facilitate local inflammation, edema, and hydrops (78, 79). This inflammatory cascade can exacerbate endolymphatic pressure and damage sensory hair cells, potentially explaining the fluctuating nature of hearing loss in MD patients after allergy (78, 80).

Mast cells respond to allergens, stress, neuropeptides, and pathogens—factors often implicated in MD flare-ups (81, 82). Research indicates a link between increased mast cell presence and various inner ear conditions (83, 84). Additionally, experimental studies have shown that triggering a type I allergic response in the endolymphatic sac of guinea pigs can produce symptoms resembling those of MD (72). Furthermore, mast cells interact with other immune and non-immune cells in the inner ear, such as macrophages and epithelial cells, creating a proinflammatory microenvironment that may perpetuate tissue damage and disrupt ion transport (85, 86).

### T cells

T cells are essential components of the adaptive immune system, orchestrating protective responses against pathogens, self-antigens, and diverse inflammatory stimuli (87). Growing evidence suggests that altered T cell responses contribute to the pathogenesis of MD (88). In particular, effector T helper subsets and cytotoxic T lymphocytes appear to play key roles in driving persistent inflammation, endolymphatic hydrops, and sensorineural hearing loss (51, 62, 89, 90).

CD4^+^ T helper cells differentiate into specialized subsets that guide immune responses through the secretion of distinct cytokines. Marioni et al. observed an increased CD4/CD8 ratio in 200 consecutive patients with MD, indicating a predominance of T-helper cells over T-cytotoxic cells (90). Th1 and Th17 subsets hold particular importance because they secrete proinflammatory cytokines such as IFN-γ, TNF-α, and IL-17 (91, 92). These cytokines activate dendritic cells and macrophages, disrupt the blood-labyrinth barrier, and recruit immune cells to the inner ear (59, 93, 94). Elevated IFN-γ and IL-17 levels in the endolymphatic sac of MD patients further underscore the role of these Th subsets in driving chronic, relapsing inflammation (42, 95). CD8^+^ cytotoxic T lymphocytes (CTLs) have also been detected in the inner ear tissues of MD patients, where they may contribute to tissue injury through direct cytolytic mechanisms (29). Lopez-Escamez et al. identified an increase in CD8^+^ memory T cells in MD patients through deconvolution of RNA-seq data from peripheral blood samples (88). These cells secrete perforin and granzymes, which trigger apoptosis in target cells, potentially leading to damage of cochlear and vestibular structures when the immune response is excessive or prolonged (79, 96). While this response may initially serve to eliminate infected or stressed cells, sustained CTL activity may lead to irreversible inner ear damage and progressive sensorineural hearing loss (97, 98).

Chronic inflammation driven by T cells not only disrupts immune homeostasis but may also result in structural changes including the inner ear (99). Persistent T cell infiltration and cytokine release can drive fibrotic remodeling, such as in the endolymphatic sac of the inner ear (100, 101), disrupt fluid regulation, and contribute to the development of endolymphatic hydrops in MD patients (27, 102). This process links immune dysregulation directly to clinical symptoms such as episodic vertigo, tinnitus, and hearing fluctuation.

### B cells

B cells are crucial players within the adaptive immune system, essential for producing antibodies, presenting antigens, and modulating immune responses through cytokine release (103). While their involvement in systemic autoimmune and inflammatory diseases is well established, their contribution to MD has received comparatively less attention (29, 104, 105). However, emerging evidence indicates that B cells could be involved in immune dysregulation in MD (88), potentially contributing to the production of autoantibodies, persistent inflammation, and tissue damage within the inner ear (29).

Histological studies of mouse temporal bone specimens have identified B cells within the endolymphatic sac (106), suggesting their active involvement in immune processes associated with MD.

B cells may play distinct roles in MD, contributing to inflammation, immune regulation, and autoimmunity. Analysis of cell clusters using the CyTOF workflow in 26 patients with definite MD revealed a significant decrease in B cells within groups expressing high levels of cytokines such as IL-1, IL-4, IL-10, IL-6, and TNF-α (29). Plasma cells, the fully matured derivatives of B cells, serve as the primary producers of antibodies (107). Elevated serum levels of immunoglobulins (IgG, IgA, and IgM) in patients with MD suggest an active humoral immune response (108). Choi et al. analyzed proteins found exclusively in the luminal fluid of patients with MD and reported that 76% were immunoglobulins or their variants, with IgM accounting for up to 41% of the total protein coverage (16). Notably, antibodies targeting various inner ear proteins are detected in 91% of sera from MD patients. These include reactivities against chicken and bovine type II collagen, the cyanogen bromide-cleaved peptide 11 (CB11) derived from type II collagen, as well as type IX and XI collagens, C-Raf, and tubulin (109). These autoantibodies may contribute to MD through multiple mechanisms, such as complement activation, leading to cytotoxic damage of cochlear and vestibular structures, and immune complex formation, which can deposit in inner ear tissues and promote inflammation and endolymphatic hydrops (17, 109).

In addition to generating antibodies, B cells influence disease development by regulating immune activity via the release of signaling molecules like IL-6 and TNF-α (29, 42, 110, 111). These cytokines are elevated in MD patients (42) and may amplify the inflammatory response in the inner ear, leading to sustained immune activation (40). Understanding the precise role of B cells in MD pathogenesis could lead to more effective, personalized treatment strategies for patients suffering from this complex inner ear disorder.

In summary, while a growing body of evidence implicates both innate and adaptive immune responses in MD, the extent and specificity of immune dysregulation remain incompletely understood. Among the most consistent findings are elevated levels of proinflammatory cytokines and increased infiltration of immune cells within the endolymphatic sac. These changes support a pathogenic model driven by chronic inflammation and tissue damage. Notably, M1-polarized macrophages and Th17 cells have been repeatedly associated with oxidative stress, epithelial barrier dysfunction, and sustained inflammation in MD. Additionally, the presence of autoantibodies targeting inner ear proteins suggests a potential autoimmune component, although their pathogenic role has yet to be definitively established. Other hypotheses, such as dendritic cell–mediated priming of autoreactive T cells or mast cell–driven allergic responses, remain less well defined and are primarily supported by correlative studies or animal models. These emerging areas warrant further mechanistic investigation. Collectively, the literature highlights that multiple immune pathways likely converge in MD, though an integrated and unified mechanistic framework has yet to be developed.

## Immune therapy for Meniere’s disease

Emerging evidence increasingly implicates immune dysregulation in the pathogenesis of MD, particularly in a subset of patients who exhibit resistance to conventional therapies (5, 112). Aberrant activation of innate and adaptive immune pathways—evidenced by elevated proinflammatory cytokines (TNF-α, IL-1β, IL-6), circulating autoantibodies, and immune cell infiltration in the endolymphatic sac—suggests an underlying autoimmune or autoinflammatory mechanism (58, 113). As such, immunomodulatory therapies are increasingly recognized as a promising approach to alleviate symptoms and potentially alter disease trajectory in MD (**Table 1**).

**Table 1:** Summary of immune-based therapies investigated in Meniere’s disease.

Therapy	Mechanism	Evidence type	Limitations
Corticosteroids (systemic or intratympanic)	Broad immunosuppression; decrease cytokines, reduce immune cell infiltration	Clinical studies, mechanistic animal data	Unclear exact mechanism in MD; variable patient response
TNF-α Inhibitors (e.g., etanercept)	Block TNF-α signaling to reduce inflammatory cascade	Case reports in autoimmune inner ear disease (AIED); experimental data	Limited MD-specific data
IL-1 Receptor Antagonist (e.g., anakinra)	Blocks IL-1-mediated inflammation	Preclinical and AIED reports	No MD clinical trials yet
B and T Cell Monoclonal Antibodies (e.g., rituximab, daclizumab)	Target lymphocytes involved in autoimmunity	AIED studies; theoretical application to MD	Immunogenicity
NLRP3 Inhibitors (small molecules)	Decrease IL-1B and IL-18 by blocking inflammasome activation	Experimental models	Preclinical stage; no MD- specific clinical data
Betahistine	Histamine H1 agonist/H3 antagonist; may decrease cytokine release	Clinical use in MD	Not a direct immunotherapy; immune effects indirect
Personalized Immunotherapy	Stratification by cytokine profiles, immune cell subsets, HLA genotyping	Conceptual, emerging studies	Biomarker discovery ongoing; implementation challenges

Corticosteroids, administered systemically or via intratympanic injection, remain the cornerstone of immune-based treatment for MD (114). These agents exert broad anti-inflammatory effects by suppressing cytokine production, downregulating adhesion molecules, and inhibiting immune cell infiltration. However, the exact mechanism by which corticosteroids alleviate symptoms in MD is not fully understood (114–117). In addition to their immunosuppressive properties, corticosteroids may influence ion transport and fluid homeostasis in the inner ear, including the regulation of Na^+^/K^+^-ATPase and aquaporin channels within the endolymphatic sac (116, 118, 119). These effects may contribute to the reduction of endolymphatic hydrops and restoration of labyrinthine function. Nevertheless, a subset of MD patients shows limited or no response to corticosteroid therapy (114), which may reflect underlying differences in immune activity, barrier integrity, or fluid regulation pathways (5, 114, 116, 120). This variability underscores the need for further mechanistic studies and individualized treatment approaches.

Beyond corticosteroids, biologic therapies targeting specific immune mediators are under investigation. Tumor necrosis factor-alpha (TNF-α) inhibitors such as etanercept, interleukin-1 (IL-1) receptor antagonists like anakinra, and monoclonal antibodies directed against B and T cell markers (e.g., rituximab and daclizumab) have demonstrated efficacy in autoimmune inner ear disease (AIED) and hold potential for MD patients with immune-mediated pathology (9, 121–123). These targeted therapies offer the advantage of modulating discrete immunological pathways while minimizing systemic immunosuppression.

Moreover, small-molecule compounds targeting the NLRP3 inflammasome, an essential component involved in orchestrating innate immune activity, have demonstrated potential in experimental models addressing inflammation within the inner ear and the nervous system (124–126). Blocking NLRP3 activation may reduce IL-1β and IL-18 secretion, thereby alleviating the inflammation underlying endolymphatic hydrops and vestibular dysfunction in MD (127, 128).

Betahistine, though not classified as an immunotherapeutic agent, is commonly prescribed for MD and may indirectly influence immune mechanisms (129, 130). It functions by stimulating histamine H1 receptors while blocking H3 receptors, thereby enhancing blood circulation in the inner ear and lowering endolymphatic pressure (129, 131). Some studies suggest that betahistine may also suppress the release of proinflammatory cytokines and modulate microvascular permeability, potentially stabilizing the blood-labyrinth barrier (132). While its primary mechanism is thought to be hemodynamic, these secondary anti-inflammatory effects may support its use in combination with immune-targeted therapies to enhance symptom control and inner ear homeostasis (5).

The advent of personalized immunotherapy offers significant potential for optimizing treatment outcomes. Stratifying patients based on immune biomarkers, such as cytokine profiles, circulating T and B cell subsets, and genetic susceptibility markers like specific HLA alleles, may enable tailored interventions that directly target the underlying immune pathology in MD (133, 134). This precision medicine approach could improve therapeutic efficacy and minimize adverse effects by identifying patients most likely to benefit from immunomodulatory treatment.

Despite recent progress, well-designed human studies remain essential to assess how safe, effective, and sustainable immune-based treatments are for MD. Advancing research efforts will benefit from combining immunophenotyping, genomic analysis, and patient-centered data to enhance treatment strategies and identify new therapeutic opportunities. As our understanding of MD immunopathogenesis deepens, immune therapy holds considerable promise for improving the management of this complex and often debilitating inner ear disorder.

## Future clinical directions and emerging questions

As our understanding of immune dysregulation in MD continues to evolve, the next frontier lies in translating mechanistic discoveries into effective, patient-specific therapies. There is an urgent need for precision immunotherapy trials that stratify individuals based on immune cell phenotypes, cytokine profiles, or genetic susceptibility markers. Advances in high-resolution technologies, such as single-cell RNA sequencing and quantitative proteomics, now provide unprecedented opportunities for biomarker discovery. These tools enable the identification of immunologically distinct subtypes of MD and support the rational selection of targeted therapies, including biologics, cytokine inhibitors, and macrophage polarization strategies (135, 136).

Several critical questions remain unanswered and should inform the direction of future research. Longitudinal immune profiling could provide insights into predicting disease exacerbations and remissions. A clearer understanding of the respective roles of resident and infiltrating immune cells within the endolymphatic sac and adjacent structures is essential for elucidating local immune dynamics. The mechanisms by which systemic immune responses influence the inner ear’s immune microenvironment remain poorly understood. Emerging evidence suggests that microbiome-immune interactions may affect disease onset, phenotype, and treatment response, potentially revealing novel targets for therapeutic intervention.

Translation of immunological findings into clinical diagnostics and therapeutics will also necessitate the development of standardized tools to measure immune activity in real-time. Potential strategies may include blood-based cytokine assays, flow cytometry panels for circulating immune subsets, and molecular imaging modalities capable of detecting inflammation in the inner ear. Furthermore, the establishment of collaborative research networks and MD-specific patient registries will be essential to enable multicenter trials, ensure diverse cohort representation, and validate immune signatures across populations.

Ultimately, progress in this field will depend on interdisciplinary collaboration. By integrating immunology with audiology, genomics, neurotology, and systems biology, the field is poised to redefine the clinical management of MD, shifting from symptom suppression to mechanism-based, disease-modifying interventions.

## Conclusion

Advancing MD research will depend on leveraging immunological insights to develop targeted, patient-specific therapies. Prioritizing translational research, biomarker discovery, and innovative clinical trials will accelerate the development of immune-guided treatments. A deeper understanding of immune cell dynamics and systemic interactions is essential to uncovering their role in disease progression.
